# Potential Role of Cellular Senescence in Asthma

**DOI:** 10.3389/fcell.2020.00059

**Published:** 2020-02-11

**Authors:** Zhao-Ni Wang, Ruo-Nan Su, Bi-Yuan Yang, Ke-Xin Yang, Li-Fen Yang, Yan Yan, Zhuang-Gui Chen

**Affiliations:** ^1^Department of Pediatrics, The Third Affiliated Hospital, Sun Yat-sen University, Guangzhou, China; ^2^Guangzhou Institute of Respiratory Diseases, The First Affiliated Hospital, Guangzhou Medical University, Guangzhou, China; ^3^Guangdong Provincial Key Laboratory of Biomedical Imaging, Guangdong Provincial Engineering Research Center of Molecular Imaging, The Fifth Affiliated Hospital, Sun Yat-sen University, Zhuhai, China; ^4^Center for Interventional Medicine, The Fifth Affiliated Hospital, Sun Yat-sen University, Zhuhai, China

**Keywords:** cellular senescence, asthma, telomere shortening, oxidative stress, senescence-associated secreted phenotype, autophagy, anti-senescence therapies

## Abstract

Cellular senescence is a complicated process featured by irreversible cell cycle arrest and senescence-associated secreted phenotype (SASP), resulting in accumulation of senescent cells, and low-grade inflammation. Cellular senescence not only occurs during the natural aging of normal cells, but also can be accelerated by various pathological factors. Cumulative studies have shown the role of cellular senescence in the pathogenesis of chronic lung diseases including chronic obstructive pulmonary diseases (COPD) and idiopathic pulmonary fibrosis (IPF) by promoting airway inflammation and airway remodeling. Recently, great interest has been raised in the involvement of cellular senescence in asthma. Limited but valuable data has indicated accelerating cellular senescence in asthma. This review will compile current findings regarding the underlying relationship between cellular senescence and asthma, mainly through discussing the potential mechanisms of cellular senescence in asthma, the impact of senescent cells on the pathobiology of asthma, and the efficiency and feasibility of using anti-aging therapies in asthmatic patients.

## Introduction

Asthma is one of the most common non-communicable pulmonary diseases. Bronchodilators and inhaled/systemic corticosteroids are the most often used drugs for asthma (Fanta, 2009). According to disease severity and symptom control assessment, patients are managed with stepwise therapy until asthma symptoms are under control. Although these standardized therapies are highly effective in most asthmatics, approximately 10% patients are of steroid-refractory (Barnes, 2013). Even with the highest step of standardized treatment, in which targeted therapies like anti-IgE, anti-IL5, anti-IL5R, and anti-IL4R antibodies would be applied, uncontrolled asthma symptoms and exacerbation still frequently exist in some patients (Israel and Reddel, 2017; Chipps et al., 2018). These patients with difficult-to-treat asthma often have higher mortality and lower lung function (McGeachie et al., 2016; Coumou et al., 2018). Furthermore, with increasing morbidity, difficult-to-treat asthma might explain the stalled reduction in global asthma mortality (Ebmeier et al., 2017). Thus, more interventions and novel strategies are in great demand for asthma patients to achieve further decrease in mortality rate. Current studies are trying to explore new mechanisms involved in the pathogenesis of asthma and then identify potential therapeutic targets.

Asthma is mainly characterized by chronic airway inflammation, airway hyperresponsiveness and airway remodeling (Papi et al., 2018). It’s a heterogenetic disease with various inflammatory phenotypes, including eosinophilic inflammation, neutrophilic inflammation, mixed inflammation, and non-inflammatory pattern (Israel and Reddel, 2017). Neutrophilic inflammation associates with the disease severity (Ray and Kolls, 2017). Structural changes like airway wall thickening and extracellular matrix deposition contribute to airway obstruction, leading to persistent airflow limitation, and reduced lung function. Thus, the abnormality of airway inflammation and alteration of airway structure constitute the basic pathophysiology of asthma.

Cellular senescence is a heterogenetic status in response to various stimuli. The main features of cellular senescence contain irreversible limitation of cell proliferation and the senescence-associated secretory phenotype (SASP), which is produced by primary senescent cells and induces senescence of surrounding cells in a paracrine manner (Nelson et al., 2012; Acosta et al., 2013). Senescent cells could be characterized by several properties, including reduced proliferative rate, increased senescence-associated β-Galactosidase (SA-β-Gal), upregulation of tumor suppressors and cell cycle inhibitors like p21, p16, p53, senescence-associated heterochromatic foci, enlarged or flat cell morphology, and secretion of multiple SASP components (Acosta et al., 2013; Muñoz-Espin and Serrano, 2014). Physiologically, cellular senescence is present during natural development and aging as a modulating mechanism, contributing to tumor suppression, wound healing (Demaria et al., 2014), and embryogenesis (Muñoz-Espín et al., 2013; Storer et al., 2013). In recent decades, increasing attentions have been addressed to its contributions to the pathogenesis of diseases and organ dysfunction. Persistent accumulation of senescent cells during aging induces low-grade inflammation through SASP (Acosta et al., 2013), impairs the immune system (Savale et al., 2009; Albrecht et al., 2014), and increases the vulnerability and susceptibility of organs to various pathological challenges (López-Otín et al., 2013). In respiratory system, cellular senescence has established role in the pathogenesis of aging-related diseases like chronic obstructive pulmonary disease (COPD) and idiopathic pulmonary fibrosis (IPF) (Tsuji et al., 2006; Diaz de Leon et al., 2010; Kuwano et al., 2016; Álvarez et al., 2017; Yanagi et al., 2017; Rashid et al., 2018; Schuliga et al., 2018; Vij et al., 2018; Araya et al., 2019; Fang et al., 2019; Parikh et al., 2019b). However, little is known about the place of cellular senescence in the development of asthma.

Amassing data has showed that pulmonary cells of COPD and IPF exhibit a senescent phenotype, which is involved in promoting airway chronic inflammation, airway remodeling, and lung function decline (Tsuji et al., 2006; Yao et al., 2012; Álvarez et al., 2017; Yanagi et al., 2017; Rashid et al., 2018; Schuliga et al., 2018; Vij et al., 2018; Araya et al., 2019; Fang et al., 2019; Parikh et al., 2019b). Increased senescence-associated proteins p16 and p21 in alveolar cells are correlated with airflow limitation of patients with emphysema (Tsuji et al., 2006). Deficiency of p21 could attenuate airspace enlargement and lung function decline in cigarette smoke-exposed mice (Yao et al., 2012). Multiple SASP components including IL-6, IL-8, TGF, and MMPs are closely associated with persistent airway inflammation and abnormal extracellular matrix remodeling or pulmonary fibrosis in COPD and IPF (Barnes et al., 2015; Richeldi et al., 2017; Álvarez et al., 2017). As asthma resembles COPD and IPF in chronic inflammation, airway remodeling as well as lung function decline (McGeachie et al., 2016; Papi et al., 2018), would it be possible that cellular senescence also promotes the development of asthma?

Limited but undeniable data has showed that cellular senescence is associated with asthma. Bronchial fibroblasts from asthmatic patients had lower DNA synthesis with cell passage and *in vitro* lifespan than normal controls (Dubé et al., 1998). Myofibroblasts from asthmatics showed reduced proliferative activity in response to mitogens *in vitro*, but higher expression of SASP factors like GM-CSF and IL-8 when stimulated by IL-1α than those from non-asthmatics (Ward et al., 2008). Asthmatic bronchial fibroblasts demonstrated greater proportion of SA-β-Gal positive staining (Hadj Salem et al., 2015). Expression of p21, a cyclin-dependent kinase inhibitor, was elevated in bronchial epithelium of asthmatics, and had a tendency to be higher in severe asthma than mild asthma (Puddicombe et al., 2003). p53 is a tumor suppression protein regulating cell proliferation and also considered as a marker of cellular senescence. It has been showed that p53 was upregulated in bronchial smooth muscle cells from asthmatics (Trian et al., 2016). Based on these evidences, we highly speculate that cellular senescence might have a similar function in asthma as it does in COPD and IPF.

In this review, we will summarize the current knowledge and research focusing on the possible involvement of cellular senescence in asthma, particularly the potential mechanisms of cellular senescence, senescent cell types and their impact on the development of asthma, as well as the effect of current and latent anti-aging strategies on asthma.

## Potential Mechanisms of Cellular Senescence in Asthma

Cellular senescence was initially discovered by Hayflick and Moorehead (1961) who described a state of cell proliferation arrest in cultured human cells after several divisions. Up to date, several stimuli causing cellular senescence have been reported, including telomere shortening due to replication exhaustion, DNA damage, mitochondrial dysfunction, oxidative stress, certain cytokines, and loss of tumor suppressor (Martínez-Zamudio et al., 2017). These factors and their downstream signal pathways constitute an intricate network leading to cell cycle arrest and SASP in target cells. Some of these stressors are found to be associated with asthma, such as telomere shortening, oxidative stress, inflammation, and autophagy.

### Telomere Shortening

Telomere shortening is one of the common mediators of cell aging and correlates to several aging-related diseases (Gansner and Rosas, 2013). Telomere shortening is generally caused by exhaustive replication and brings about cell cycle arrest (Hayflick and Moorhead, 1961). Telomeres locate at the end of chromosomes in mammalian cells and gradually shorten after each round of cell division because they remain unduplicated during DNA synthesis phase. When they reach the critical length, the ability of cell division will be restricted (Nikitina and Woodcock, 2004).

Scientists have observed decreased proliferation of bronchial fibroblasts and myofibroblasts in asthmatic subjects as compared to non-asthmatics, despite the apparent thickening of airway smooth muscle layer and high levels of inflammatory factors, and suggesting a premature status of cellular senescence in asthma (Dubé et al., 1998; Ward et al., 2008). To explore the underlying mechanism of cell replicative restriction in asthmatics, Hadj Salem et al. (2015) measured the telomere length in bronchial fibroblasts from asthmatic patients and healthy controls. They observed decrease of telomere length in asthmatic fibroblasts, correlating with the increase of the cellular senescence marker β-Galactosidase (Hadj Salem et al., 2015). Similarly, Lee et al. (2017) also found that relative telomere length in peripheral blood mononuclear cells (PBMCs) was shorter in asthmatic children and adolescences than that of non-asthmatics. Likewise, leukocytes from asthmatic patients seemed to have shorter telomere length compared to age-matched controls (Kyoh et al., 2013; Belsky et al., 2014). Shorter telomere length has been proposed as a biomarker of accelerating aging (López-Otín et al., 2013; Bernadotte et al., 2016). These evidences implicate the existence of cellular senescence of bronchial structure cells and immune cells of asthmatics.

Telomere shortening may not only explain the limited proliferation ability of cells in asthmatic patients, but also highly correlated to the clinical features and severity of asthma. For example, telomere shortening in bronchial fibroblasts was associated with airway hyperresponsiveness (Hadj Salem et al., 2015) and lower forced expiratory flow (Henckel et al., 2018). Furthermore, shorter telomere served as a biomarker of life-course-persistent asthma and was linked to eosinophilic inflammation (Belsky et al., 2014). Decrease of telomere length in bronchial fibroblasts was associated with increased severity of asthma (Kyoh et al., 2013). Telomere length may also reflect therapeutic effect for asthma. Asthmatic patients who received steroid treatment would have less telomere shortening than those did not (Lee et al., 2019). These results show that telomere shortening might be a critical biomarker correlating to the pathophysiology of asthma.

Although studies have described the closely association between asthma and telomere shortening, the controversy is that whether shorter telomere accelerates the development of asthma, or telomere shortening is resulted from asthma? According to a study, life-course-persistent asthma, along with higher eosinophilic inflammation, correlated with shorter telomere length than childhood-onset, adulthood-onset asthma, and no-asthma controls (Belsky et al., 2014). One possibility is that higher eosinophilic inflammation promotes telomere shortening. However, the authors found that there was no rapid change of telomere length between age 26 and age 38 in patients with life-course-persistent asthma, indicating that eosinophilic inflammation has little effect on the telomere shortening (Belsky et al., 2014). Thus, telomere shortening is more likely to be a cause of greater inflammation in asthma, rather than a result.

Various factors like environmental pollutants and lifestyle could influence the telomere length from young age (Mirabello et al., 2009; Cassidy et al., 2010). Studies have shown that the rate of telomere shortening accelerated in children exposed to air pollutants such as polycyclic aromatic hydrocarbons (PAHs), ozone (O_3_) and fine particulate matter (PM_2_._5_) (Lee et al., 2017, 2019). Cigarette smoke exposure also has an adverse effect on the telomere length in children (Ip et al., 2017). Air pollutants and cigarette smoke are common extrinsic inducers to evoke oxidative stress and inflammation in the airway (Zhang X. et al., 2016; Chandrasekaran et al., 2017), leading to the vulnerability of telomere (Von Zglinicki, 2002; Venkatachalam et al., 2017). Prenatal stressor like higher tumor necrosis factor-α/interleukin 10 (TNF-a/IL-10) ratio could also lead to shorter telomere length in newborns (Lazarides et al., 2019). Nuclear factor kappa-B (NF-kB)-driven chronic inflammation could accelerate the rate of senescence in mice through enhancing the expression of cyclooxygenase-2 (COX-2) and reactive oxygen species (ROS), which promote DNA damage and telomere dysfunction (Jurk et al., 2014).

Although some of these stressors are involved in the development or exacerbation of asthma (Guarnieri and Balmes, 2014; Underner et al., 2015), whether telomere shortening induced by these childhood or prenatal risk factors would lead to asthma or not is still obscure. Suh et al. (2019) followed up 84 subjects and found that higher prenatal stress and shorter telomere length did not increase the risk of developing preschool asthma. Thus, more prospective and large-sample investigations are required to answer this question.

### Oxidative Stress

Oxidative stress is long been considered as an inducer of premature senescence (Finkel and Holbrook, 2000). Various oxidants like peroxide hydrogen (H_2_O_2_), which is also an endogenous oxidant, are commonly used to trigger stress-induced premature senescence in experimental studies (Wang et al., 2013). Oxidative stress can be manifested into increased ROS production and decreased antioxidant capacity within the cells (Thannickal and Fanburg, 2000). ROS overproduction could result from mitochondrial oxidative metabolism (Chandel, 2010), respiratory burst, and exposure to environmental pollutants like O_3_ and cigarette smoke (Chandrasekaran et al., 2017). Oxidative stress is aggravated during chronic inflammation, due to the release of ROS by multiple immune cells including activated neutrophils, macrophages, monocytes, and dendritic cells (Sánchez et al., 2015). Thus, allergens, environmental noxa, and inflammatory factors act on cells, resulting in altered function of mitochondria, elevated production of ROS, and then perpetuate inflammation as a positive feedback loop. The formation of this vicious cycle will finally lead to SASP by activating nucleotide-binding domain, leucine-rich repeat-containing family protein (NLRP)-3 inflammasome and releasing inflammatory cytokines such as TNF-α, IL-1β, IL-6, and IL-18 (Davalli et al., 2016).

Evidences have revealed the role of oxidative stress in the pathogenesis of asthma (Bullone and Lavoie, 2017; Kleniewska and Pawliczak, 2017). The ROS in asthma could come from resident cells or immune cells. Mixed allergens could significantly induce ROS production in airway epithelial cells *in vitro* with the absence of immune cells (Chan et al., 2016, 2017; Chen J. et al., 2017). Pollutants like PM_2_._5_ could also promote ROS production in human lung alveolar epithelial A549 cells (Deng et al., 2013). ROS production is closely associated with neutrophilic and Th17 inflammation, which are involved in the development of asthma (Chesné et al., 2014; Ray and Kolls, 2017; Carr et al., 2018), and correlated to exacerbation and asthmatic patients with obesity (Suzuki et al., 2008; Kim et al., 2014; Ray and Kolls, 2017; To et al., 2018). Elevated ROS generation from neutrophils and macrophages in asthmatic subjects is correlated to increase of NLRP3 inflammation (Simpson et al., 2014), leading to airway hyperresponsiveness, and lung fibrosis (Kim et al., 2014; Sun et al., 2015).

The mechanism of cellular senescence induced by oxidative stress is involved with a complicated process. Chan et al. (2016, 2017) demonstrated that HDM challenge could enhance ROS generation and elevate the expression of DNA-damaging marker γH2AX. At the same time, DNA repair associated protein was also upregulated (Chan et al., 2016, 2017). The former response would lead to cell cycle arrest and cell death, while the latter could result in cell survival. Cellular senescence might be an intermediated state resulted from the conflict of oxidative stress-induced DNA damage and DNA repair, because senescent cells are still alive but with proliferation arrest (Hayflick and Moorhead, 1961). Probably these affecting cells are not killed because of insufficient DNA damage, and they stop cell diving due to inadequate DNA repair. From another perspective, exogenous and endogenous sources of ROS in asthma could simultaneously activate multiple signaling pathways, including NF-κB, p53, phosphoinositide-3-kinase (PI3K)/protein kinase B (Akt) and p38 mitogen-activated protein kinases (MAPK) (Finkel and Holbrook, 2000). p53 serves as a checkpoint protein and its downstream factor p21, a cell cycle dependent kinase inhibitor, could lead to cell cycle arrest (Surget et al., 2013). However, PI3K/Akt/mammalian target of rapamycin (mTOR) pathway could induce chronic inflammation, inhibit cell death, and promote cell proliferation (Bent et al., 2016). Their combinational effect finally brings about a senescent state in cells. This theory has been proved by a previous investigation, which demonstrated that both cell cycle blockage and growth stimulation were required for the development of cellular senescence (Demidenko and Blagosklonny, 2008).

### Inflammation

Chronic inflammation serves as the principal hallmark of asthma. Previous studies had shown that aged people with asthma would have higher inflammation levels, which contributed to the therapy unresponsiveness (Busse et al., 2017; Dunn et al., 2018). Intimate association between senescence and inflammation has been depicted in various diseases, such as COPD, inflammatory bowel disease (IBD), cardiovascular disease, obesity and diabetes, autoimmune diseases, and cancer (Zhang J. et al., 2016). According to current understanding, the interrelationship between inflammation and cellular senescence is mainly mediated by the SASPs (Fougère et al., 2017).

Senescence-associated secreted phenotype was firstly defined by Coppé et al. (2008) in and now has been considered as a hallmark of cellular senescence. They found that these secretory phenotypes formed only after DNA damage in fibroblasts and epithelial cells (Coppé et al., 2008). SASPs include inflammatory cytokines such as interleukin-6 (IL-6), interleukin-8 (IL-8) and monocyte chemoattractant protein-1 (MCP-1), growth regulators such as GRO and insulin-like growth factor binding protein-2 (IGFBP-2), cell survival modulators such as OPG and sTNF RI, and shed surface proteins such as uPAR and ICAM-1. Although the SASP in senescent fibroblasts and epithelial cells are not totally the same (Coppé et al., 2008), they execute similar functions in lung diseases, such as promoting cellular senescence, wound repair, and airway remodeling (Parikh et al., 2019b).

Senescence-associated secreted phenotypes reflect an active but abnormal metabolic state of senescent cells despite of quiescence in cell proliferation (Zhang J. et al., 2016). Since 1998, researchers had found that even though lung fibroblasts in asthmatics decreased in proliferation capability, they were still active in producing extracellular matrix proteins such as collagen (Dubé et al., 1998; Ward et al., 2008). SASP is primarily a DNA damage response (DDR) (Rodier et al., 2009). Its secretion is mediated by intracellular IL-1α/miR-146a/b/IL-6/C/EBP-β loop and p38/NF-κB and mTOR pathways (Tchkonia et al., 2013). Senescent cells have a bystander effect on the nearby healthy cells. Co-culture with senescent fibroblasts could increase the generation of DNA double-strand breaks (DSBs) foci in young fibroblasts, indicating that senescent cells could induce DDR in surrounding proliferating cells (Nelson et al., 2012). Soluble factors released into the culture medium from senescent cells hardly promote DNA damage in young cells, but they can transmit to the attached surrounding cells via cell-cell junctions. Blocking these gap junctions would attenuate the increase of DNA damage foci (Nelson et al., 2012). Some components of SASP could induce cellular senescence in a receptor-mediated manner. For example, Jin et al. (2016) found that MCP-1, one of the dominant components of SASP, enhanced senescence in mesenchymal stromal cells (MSCs) via activating the cognate receptor chemokine (c-c motif) receptor 2 (CCR2) and its downstream ROS-p38-MAPK-p53/p21 signaling cascade. Activated p53 would then elevate secretion of MCP-1 to form a positive feedback loop (Jin et al., 2016).

By now, it is still hard to exactly distinguish whether intrinsic inflammation in asthma induces cellular senescence or senescent cells result in airway chronic inflammation *via* SASP. On one hand, some stressors can lead to low-grade inflammation in the airway before asthma onset. Exposure to HDM extract could induce the upregulation of several components of SASP, such as MCP-1, IL-6 and IL-8 in human monocytes (Lee et al., 2008). Childhood obesity is a risk factor for asthma. Postnatal hyperalimentation could enhance the expression of inflammatory cytokines including IL-6, TNF-α, and IL-17A and then induced airway hyperresponsiveness in mice (Dinger et al., 2016). On the other hand, preexisting asthmatic inflammation has different effect on cellular senescence. TSLP plays an important role in inducing Th2 inflammation and airway remodeling in asthma (Soumelis et al., 2002; Chen et al., 2013). Wu et al. (2013a) found that TSLP could trigger senescence in airway epithelial cells *in vitro*, indicated by the upregulation of p21, p16 and SA-β-Gal. However, Belsky et al. (2014) conducted a prospective study and demonstrated that higher eosinophilc inflammation did not accelerate telomere shortening rate in asthma. Thus, cellular senescence in asthma might be only induced with some specific inflammatory factors.

### Autophagy

Autophagy is an intercellular self-degradation process responding to various stimuli including inflammation, pathogenic infection, environmental pollutants, and hypoxia to maintain cellular homeostasis. The role of autophagy in cellular senescence is quite debated, because both its activation and inhibition effects have been reported. Kang and Elledge (2016) suggested that there are two kinds of autophagy: selective autophagy and general autophagy. In selective autophagy, specific components rather than global bulk would be cleaned out through receptor-mediated phagocytosis into autophagosome or lysosome. Selective autophagy could suppress cellular senescence by degrading GATA4, which could initiate NF-κB pathway and induce SASP. On the contrary, general autophagy would promote senescence through TOR-autophagy spatial coupling compartment (TASCC) to facilitate the production of SASP-associated factors (Kang and Elledge, 2016).

Autophagy has been implicated in asthma pathogenesis, but whether it serves as a protective or promoting role is also controversial. Genetic variants of autophagy gene 5 (ATG5) have been found to correlate with asthma exacerbation (Martin et al., 2012) and prebronchodilator FEV_1_ in asthmatic patients (Poon et al., 2012). In patients with severe asthma, the level of autophagy in peripheral blood cells and eosinophils is higher than that in non-severe asthma or healthy controls (Ban et al., 2016). McAlinden et al. (2019) found that autophagy is activated in HDM-induced asthma mice with increased Beclin 1 and ATG5 in airway epithelium and airway smooth muscle, and autophagy inhibitor chloroquine could significantly reduce airway inflammation, hyperresponsiveness, and structure remodeling. Conversely, another study showed that autophagy stimulator Simvastatin could alleviate Th2 inflammation and extracellular matrix deposition in asthmatic mouse model (Gu et al., 2017). Suzuki et al. (2016) also discovered that ATG5-mediated autophagy could attenuate airway hyperresponsiveness and neutrophil inflammation, while ATG5 depletion would lead to development of glucocorticoid resistance, and severe IL17A-dependent neutrophil inflammation.

The relationship between cellular senescence and autophagy in asthma is also vague. In respiratory system, it was proposed that insufficient autophagy or mitophagy would induce cellular senescence in COPD and IPF (Fujii et al., 2012; Ito et al., 2015; Kuwano et al., 2016; Tsubouchi et al., 2018; Vij et al., 2018; Araya et al., 2019). For example, insufficient autophagy of mitochondria would increase ROS production and therefore lead to oxidative stress (Gomes and Scorrano, 2013). P62 could combine with polyubiquitinated substrates and Atg8/LC3 to form an important component of autophagosome, and regulate the delivery of ubiquitinated proteins for selective autophagic degradation. Thus, cumulative p62 and ubiquitinated proteins are thought to be the indicators of insufficient autophagy (Komatsu et al., 2007). Transient and insufficient activation of autophagy induced by cigarette smoke extract (CSE) lead to the accumulation of p62 and ubiquitinated proteins, resulting in increased cellular senescence and SASP in human bronchial epithelial cells (HBECs) (Fujii et al., 2012; Ito et al., 2015). Sufficient autophagy activated by Torin1 could avoid amassing of p62 and ubiquitinated proteins, and therefore prevent developing into cellular senescence (Fujii et al., 2012). In asthma, p62 plays a pivotal role in mediating Th2 inflammation in allergic airway diseases (Martin et al., 2006). Although p62 was decreased in airway epithelium and smooth muscle layer of HDM-induced asthma mice (McAlinden et al., 2019), it was upregulated in CD11c^+^ cells to promote higher neutrophilic airway inflammation and hyperreactivity (Suzuki et al., 2016). Suzuki et al. (2016) found that impaired autophagy in CD11c^+^ cells, but not in epithelial cells, contribute to severe airway inflammation and steroid resistance. Therefore, the effect of autophagy on asthma depends on not only the kind of autophagy, but also the type of target cells.

According to the previous studies, insufficient autophagy and non-selective autophagy might contribute to cellular senescence (Fujii et al., 2012; Kang and Elledge, 2016). However, most of current investigations have neglected the type of autophagy when studying the correlation of autophagy and asthma. Autophagy is a complicated biological process so that it’s difficult to figure out which type it is. In order to identify the function of autophagy on asthma, probably an easier way is to detect whether the affected cells are senescent or not. The link of autophagy, cellular senescence and asthma should be identified. Thus, it is necessary for future studies to pay attention to the type of autophagy, the type of target cells and the outcome of the affected cells.

## Senescent Cells and Their Influences on the Development of Asthma

### Epithelial Cell Senescence

Airway epithelium physiologically functions as the first line of defense in innate immunity, preventing intrusion of extraneous particles such as pathogens, allergens, and environmental pollutants from inhaled air into lung. In asthma, epithelial cells were damaged and functioned abnormally by promoting pathologically tissue repair and inducing chronic airway inflammation through the release of cytokines like TSLP, IL-25 and IL-33 (Lambrecht and Hammad, 2012; Gon and Hashimoto, 2018; Papi et al., 2018). Epithelial senescence plays a pivotal role in the initiation of chronic airway diseases. In COPD and IPF, senescence of airway epithelial cells is mainly mediated by mitochondrial dysfunction and DNA damage (Mora et al., 2017; Zhang et al., 2017; Fang et al., 2019). In aged people, the barrier function of airway epithelium was impaired, making them more vulnerable to infections, which could initiate the exacerbation of chronic diseases such as asthma and COPD (Boulet et al., 2017; Yanagi et al., 2017). Telomere shortening and cellular senescence in type II alveolar epithelial cells (AECs), rather than mesenchymal cells such as myofibroblasts, resulted in airway remodeling and lung fibrosis (Naikawadi et al., 2016). Besides, increased inflammatory cell infiltration in the bronchoalveolar lavage fluid (BALF) was accompanied with higher senescent type II AECs in telomere repeat binding factor 1 (TRF1) -depleted mice (Naikawadi et al., 2016). Thus, senescence in airway epithelial cells plays a key role in initiating airway remodeling and inflammation.

Although there is only a few direct evidence showing senescent epithelial cells in the lung tissues of asthmatic patients (Puddicombe et al., 2003), one study has proved that TSLP could induce cellular senescence in airway epithelial cells *in vitro* (Wu et al., 2013a). Low dose exposure of air pollutants PM_10_ could lead to airway inflammation through inducing oxidative stress and mitochondrial dysfunction (Chan et al., 2019), which has been implicated to result in epithelial cell senescence (Tezze et al., 2017). Epithelium senescence might promote asthma development through damaging the epithelial integrity and barrier function. ITGB4 is a critical structural adhesion molecule maintaining the integrity of airway epithelium. One study found that ITGB4 expression was downregulated in OVA-challenged mice accompanied with reduced wound repair ability and anti-oxidant capacity (Liu et al., 2010a). ITGB4 was also found to be decreased in asthmatic patients (Liu et al., 2010b). Deficiency of ITGB4 could result in cellular senescence in airway epithelial cells through p53 signaling pathway (Yuan et al., 2019). Besides, ITGB4 deficiency could also result in severe airway inflammation and airway hyperresponsiveness in asthma (Liu et al., 2010a). Thus, epithelial cell senescence induced by the downregulation of ITGB4 or increased TSLP leading to airway epithelium dysfunction, might be an important mechanism of asthma pathogenesis. However, more evidences are needed to further certify that epithelial cell senescence initiates the development of asthma.

### Mesenchymal Cell Senescence

Mesenchymal cells in the airway include lung fibroblasts, myofibroblasts and airway smooth muscle cells (ASMCs). Fibroblasts might be the most commonly used model for studying cellular senescence. Previous studies have detected premature senescence of bronchial fibroblasts and myofibroblasts in asthmatic lungs (Dubé et al., 1998; Ward et al., 2008; Hadj Salem et al., 2015). SASP-related cytokines, chemokines, matrix-remodeling proteases expressed by senescent lung fibroblasts could result in low-level inflammation and fibrosis (Schafer et al., 2017; Álvarez et al., 2017). Clearance of these senescent fibroblasts by senolytic drugs would render the resolution of fibrosis (Schafer et al., 2017). The effect of fibroblast senescence on the pathobiology of asthma is not clear yet, but we could still find some clues. Some investigators have demonstrated that the activation of transcription factor signal transducer and activator of transcription 3 (STAT3) might contribute to lung fibroblast senescence in patients with IPF (Waters et al., 2018, 2019). They found that nuclear localization of STAT3 was elevated in senescent fibroblasts while inhibition of STAT3 activity would attenuate the accumulation of SA β-Gal and mitochondrial dysfunction. STAT3 plays a vital role in lung inflammation and airway remodeling in asthma, and it has been well proved as the downstream signal of TSLP (Wu et al., 2013b; Gavino et al., 2016). Therefore, the activation of STAT3 in asthma may induce chronic inflammation and airway remodeling via promoting lung fibroblast senescence. Further evidences are still needed to confirm the role of fibroblast senescence in airway inflammation and remodeling of asthma.

Airway smooth muscle cells is another integral cell type constituting the airway structure. One featured symptom of asthmatic patients is airway hyperresponsiveness, which is mainly induced by the contraction of ASMCs in response to specific stimuli. Persistent chronic inflammation and secreted growth factors could lead to increased airway smooth muscle mass and then promote irreversible airway obstruction. Some asthma-related factors have shown to induce senescence in smooth muscle cells. *In vitro* study showed that hypoxia induced cellular senescence in fetal ASMCs, leading to the upregulation of proinflammatory and profibrotic mediators, as well as increased contractility, which conduces to inflammation, tissue remodeling and airway obstruction (Parikh et al., 2019a). IgE and its receptor play an important role in the pathogenesis of allergic diseases like asthma. Recent study found that IgE induced senescence of smooth muscle cells via upregulating lincRNA-p21 and p21 in OVA-asthma model (Guo et al., 2019).

However, it is unclear whether ASMCs from asthmatics are senescent or not. Some studies suggested that the proliferation rate of ASMCs from asthmatic subjects was enhanced (Johnson et al., 2001; Trian et al., 2016). Although Trian T and coworkers found that senescent marker p53 was increased in asthmatic ASMCs, it seems that p53 had lost its anti-proliferative function in asthma (Trian et al., 2016). On the other hand, some research failed to detect the increase of nuclei numbers or proliferative markers like Ki67 in airway muscle bundles of asthma (Benayoun et al., 2003; Moir et al., 2003). By using bronchial biopsies from 14 subjects with mild to moderate asthma and 15 control subjects, Woodruff et al. (2004) demonstrated that there was hyperplasia but not hypertrophy in smooth muscle. Conversely, with a larger sample (about 50 subjects per group), James et al. (2012) found that ASMCs hypertrophy was present in both fatal and non-fatal asthma while hyperplasia only occurred in fatal asthma. In acute asthmatic murine model, Ki67 was upregulated in ASMCs. However, in chronic model (with significant airway remodeling), ASMCs exhibited hypertrophic cell shape instead of increased proliferation rate (Plant et al., 2012). Thus, persistent course of asthma or severe asthma might result in ASMC hypertrophy instead of hyperplasia. Furthermore, increased oxidative stress burden in asthma is also more likely to induce hypertrophy of ASMCs. Genome-wide microarray analysis identified increased expression of NADPH oxidase (NOX) subtype 4 (Nox4) in primary airway smooth muscle of asthma (Sutcliffe et al., 2012). TGF-β1 could also promote human ASMC hypertrophy through inducing Nox4 expression (Sturrock et al., 2007). Interestingly, Nox4 overexpression could not only induce hypertrophy of vascular smooth muscle cells (VSMCs) but also lead to stronger SA-β-Gal staining (McCrann et al., 2009). Besides, Zhou et al. (2004) found that increased expression of p21 could result in hypertrophy and cell cycle arrest in human ASMCs. With respect to these reports, it’s highly possible that hypertrophic ASMC is a senescent phenotype as it has enlarged cell morphology and proliferation suspension, but future studies still need to use more senescence-associated markers to identify whether hypertrophic ASMCs are senescent or not.

### Immune Cell Senescence

The third cell type that might suffer from cellular senescence in asthmatics is immune cell. As we’ve mentioned above, PBMCs and leukocytes from asthmatic patients have experienced telomere shortening, which is one of the characteristics of cellular senescence (Kyoh et al., 2013; Belsky et al., 2014; Lee et al., 2017). In clinical studies, immune cells isolated from peripheral blood might be the most commonly used subjects for studying cellular senescence in diseases as they are easy-obtained. Accelerated aging of leukocytes from asthmatics patients was associated with longer course of disease (Belsky et al., 2014). Brandenberger and Mühlfeld (2017) concluded that immune senescence in aged people impaired both innate and adaptive immunity, making organisms more susceptible to infection, and contributing to the development of chronic lung diseases. Affected immune cells in the elderly may include macrophages, neutrophils, natural killer (NK) cells, dendritic cells, B cells and T cells, leading to higher levels of IL-6, IL-8, and TNF-α. The inflammatory response was more severe while the ability of pathogen clearance declined. Furthermore, senescence of T cells would also alter the T-cell mediated immunity and its regulatory immune function, facilitating the development of autoimmune diseases (Lynch et al., 2017).

Most of the studies related to immune senescence were reported in aged people, while there are still a few studies that reveal the effect of immune senescence on the disease development in young people. Balint et al. (2013) presented a premature immune senescence in multiple sclerosis (MS) children who had lower numbers of naive T cells as well as reduced recent thymic emigrants of Treg cells compared to their healthy counterparts, indicating the impairment of T cells hemostasis. This finding may provide further support to the relationship of immune senescence and pathogenesis of asthma, with which some patients exhibit Th17/Treg bias (Carr et al., 2018; Papi et al., 2018). Th17/Treg bias is more common in aged people with asthma. The Th17/Treg ratio rises up with aging and contributes to a proinflammatory status (Schmitt et al., 2013). Th17 cells differentiated from naïve T cells when stimulated by IL-6 and TGF-β, which are common components of SASP. This might be the reason of why Th17 cells increase along with aging. Interestingly, IL-17 could also enhance the secretion of SASP cytokines in bronchial fibroblasts, such as GM-CSF, TNF-α, IL-1β, and IL-6 (Molet et al., 2001). Though Treg cells also increase with aging, its function to suppress Th17 cell expansion is deficient (Jagger et al., 2014). Th17/Treg bias in asthma, probably affected by immune cell senescence, contributes to neutrophil inflammation, and difficult-to-treat phenotype (Papi et al., 2018). Recent study found that it is impaired autophagy of immune cells, but not epithelial cell, contributed to severe Th17-mediated neutrophil inflammation, and steroid resistance (Suzuki et al., 2016). Impaired autophagy is thought to trigger cellular senescence (Fujii et al., 2012), thus this study indicates that immune cell senescence might have greater contribution to chronic airway inflammation in asthma.

## Therapeutic Role of Anti-Aging Strategies in Asthma

Though the role of cellular senescence in the asthma development is still under investigation, some of the anti-aging strategies have been proven to improve the airway inflammation or airway remodeling in asthma. According to our previous discussion, senescence-associated triggers like telomere shortening, oxidative stress, inflammation, and autophagy are greatly associated with the development of asthma. Thus, the therapy purpose is to inhibit these potential mediators. As current therapies for asthma have reached an impasse, anti-senescence strategies might provide a new perspective for asthma treatment.

To avoid natural aging, multiple strategies have been put forward, including healthy lifestyle, caloric restriction and weight loss in obese, as well as some pharmacological interventions (de Cabo et al., 2014). Some drug candidates have been introduced, such as azithromycin, metformin, resveratrol, rapamycin, and roxithromycin. Although their underlying mechanisms are undetermined, it’s proposed that they could block aging progress *via* direct or indirect activation of autophagy in target cells (de Cabo et al., 2014). Most of the anti-aging drugs are repurposing from existing drugs, with the advantage of being thoroughly screened for safety and clear mechanisms of action (Snell et al., 2016). Here we will describe the current anti-senescence therapies that might be effective for improving asthmatic symptoms and pathobiology.

### Caloric Restriction and Weight Loss

Previous reports showed that intermittent fasting or calorie restriction might improve immune function and ameliorate inflammation in some conditions (Buono and Longo, 2019; Collins et al., 2019). These two studies demonstrated that cycled fasting would help inhibit inflammation response to various stimuli and elevate the anti-infection effect of immune system. For example, normal mice needed to spend 1 week to totally clear off the invasive pathogens, while fasting mice just consumed 2 days. Furthermore, inflammation level was reduced in adults after fasting for 19 h. This is of importance because inflammation is a double-edged sword. Persistent or excessive inflammation would facilitate the development of various chronic diseases or cancer, as well as induce cellular senescence and organ aging. Thus, caloric restriction and weight loss have been proposed to be one of the efficient interventions to delay aging process (de Cabo et al., 2014). Although the mechanism involved is not fully understood, caloric restriction may prevent cellular senescence through eliminating inflammation- and oxidative-induced damage and activates selective autophagy to remove the present damage components (Fontana et al., 2018). Besides, calorie restriction can also ameliorate the circulating insulin growth factor 1 (IGF-1) level and mTOR activation, which could lead to premature senescence in cells (Fontana et al., 2018). Asthma coexistence with obesity tends to become more severe and difficult-to-treat (Kim et al., 2014; To et al., 2018). To et al. (2018) demonstrated that obesity-derived oxidative stress was to blame for the asthma outcomes. Obesity is more associated with Th17 and neutrophilic inflammatory phenotypes, leading to NLRP3 inflammasome activation (Kim et al., 2014), which would induce cellular senescence and SASP (Davalli et al., 2016). Johnson et al. (2007) found that after alternate days of calorie restriction, serum levels of oxidative substances and inflammation were reduced and the levels of antioxidant uric acid were increased in obese people with asthma. A systematic analysis also concluded that caloric restriction and weight loss were beneficial for disease control, lung function and life quality in asthmatic patients (Forte et al., 2018). However, whether caloric restriction exerts similar effect in asthmatic patients with normal weight is unknown.

### Senolytic Drugs

Majority of senolytic agents are selected from FDA-approved drugs and repurposed through *in vitro* or *in vivo* senescent models. Although senolytic drugs are still in their infancy in clinical trials, some drugs with potential anti-aging effect have been proved to be medicative in asthma. Azithromycin (AZM), a 15-membered macrolide originated from erythromycin, is not only with bactericidal effect, but also deemed to be anti-inflammatory and capable of regulating inflammatory response (Kanoh and Rubin, 2010). Clinical benefits in asthma contain improvements of peak expiratory flow, symptoms and life quality (Reiter et al., 2013; Gibson et al., 2017). AZM has been shown previously to strengthen the airway epithelial barrier and therefore decrease the invasion of inhaled allergens and pathogens (Slater et al., 2016). Recently, Ozsvari et al. (2018) identified AZM as a novel senolytic drug to clean about 97% of senescent human lung fibroblasts *in vitro*, indicating that AZM might help remove senescent fibroblasts and reduce SASP-related factors in asthmatic lungs, and then attenuate airway inflammation and airway remodeling. According to this study, the senolytic activity of AZM might be through inducing selective autophagy to preferentially target senescent cells and accelerate their death. Metformin, a widely used hypoglycemic drug, has been demonstrated to improve the clinical outcomes of patients with coincidence of asthma and diabetes when compared with placebo controls (Li and Li, 2016; Wu et al., 2019). However, such results did not happen in the patients using insulin (Chen C. Z. et al., 2017), indicating that metformin may achieve this effect through other mechanism instead of just lowering blood glucose level. Metformin is also known as senolytic drug candidate, suggested to activate AMP-activated protein kinase (AMPK), the upstream regulator of autophagy, to protect cells against apoptosis and senescence (Chen et al., 2016; Garg et al., 2017). Activation of AMPK by metformin could attenuate CSE-induced inflammation in airway epithelial cells and elastase-induced airspace enlargement. This effect is probably through metformin’s senolytic activity as it could reduce the expression of senescence-related genes such as p21 and p16, as well as SASP components like IL-6, IL-8, and MCP-1 in CSE-treated epithelial cells and elastase-stimulated mice (Cheng et al., 2017). SASP not only contributes to chronic inflammation, but also is involved in airway remodeling via expression of profibrotic factors and extracellular matrix (Parikh et al., 2019b). Park et al. (2012) found that metformin could reduce eosinophilic inflammation and peribronchial fibrosis, smooth muscle layer thickening, and mucin secretion though activating AMPK and decreasing oxidative stress in murine model of chronic asthma. However, further investigations need to ascertain if metformin is through inhibiting SASP and eliminating senescent cells in asthma to attenuate airway inflammation, ECM deposition and airway wall thickening. Other senolytic drugs including resveratrol, rapamycin and roxithromycin also have been demonstrated to protect asthmatic patients from persistent airway inflammation and airway remodeling, such as attenuating airway fibrosis and reducing bronchial smooth muscle mass (Shimizu et al., 1994; Black et al., 2001; Chen et al., 2015; Hua et al., 2015; Wu et al., 2015). These data give us a new insight into therapeutic role of senolytic drugs in treatment of asthma. Besides, their medicative effect on asthma also provides additional evidences to suggest the role of cellular senescence in the pathogenesis of asthma.

### Stem Cells Transplantation

Remarkable experimental and clinical trials have demonstrated the therapeutic effect of mesenchymal stem cells (MSCs) in the diseases of various organs or systems with the capabilities of regeneration and immunomodulation (Uccelli et al., 2008). In recent years, with consideration of their merits of cell replacement and improving airway microenvironment, increasing clinical studies of intravenous injection of MSCs to patients with aging-related diseases including aging frailty, cardiovascular diseases, IPF and COPD have been conducted (Weiss et al., 2013; Golpanian et al., 2016; Bartolucci et al., 2017; Glassberg et al., 2017; Tompkins et al., 2017). MSCs transplantation was shown to improve the physical performance, immune function, FEV_1_ and quality of life in patients with aging frailty, which was featured by exhaustion of stem cells or precursor cells, and chronic inflammation (Golpanian et al., 2016; Tompkins et al., 2017). In patients with aging-related respiratory diseases such as COPD and IPF, MSCs infusion would alleviate inflammation and lung fibrosis (Royce et al., 2014). In murine asthma models, intravenous injection of MSCs would suppress inflammatory cells infiltration and cytokines secretion, and ameliorate histopathological changes (Bonfield et al., 2010; Firinci et al., 2011). MSC exosomes could promote proliferation of Treg cells to restore Th17/Treg homeostasis in aged people and some difficult-to-treat asthmatics through its immunosuppression effect (Du et al., 2018). MSCs infusion could also downregulate the expression of SASP-associated cytokines (TNF-α, IL-1β, MCP-1, and IL-6) and proteases (MMP9 and MMP12) in lung with cigarette smoke exposure (Guan et al., 2013), indicating that MSCs play an important role in ameliorating SASP. However, whether stem cell therapy could selectively clean out the senescent cells is not yet demonstrated. Thus, Further studies need to detect the change of senescence-associated markers in asthmatic patients after MSCs administration.

## Specific Role of Cellular Senescence in Asthma Different from COPD and IPF

The contribution of cellular senescence in the pathogenesis of COPD and IPF is quite well established. Similar to COPD and IPF, asthma is also initiated from airway epithelium injury, sharing the analogical pathobiology features including chronic airway inflammation and airway remodeling. Thus, based on the facts that cellular senescence could promote chronic inflammation and airway fibrosis in COPD and IPF, and cellular senescence could be detected in asthmatic subjects (Dubé et al., 1998; Puddicombe et al., 2003; Ward et al., 2008; Hadj Salem et al., 2015), we highly propose that cellular senescence is a potential mechanism for asthma development.

However, some characteristics of asthma are different from COPD and IPF, suggesting that cellular senescence might be triggered or effect differently in this disease. First of all, asthma can be child-onset, while COPD and IPF are often diagnosed at adulthood and old age. This indicates that there is premature senescence triggered at young age in asthma. In this regard, studies have demonstrated that hypoxia in infants, air pollutants exposure, and allergen challenge in childhood/adolescence could induce underlying senescence process (Deng et al., 2013; Chan et al., 2016, 2017; Lee et al., 2017, 2019; Parikh et al., 2019a). Such stressors might induce cellular senescence in airway epithelial cells, mesenchymal cells or immune cells, leading to their dysfunction and facilitating the initiation of asthma. Second, asthma is a heterogenetic disease with various inflammation phenotypes such as eosinophilic, neutrophilic, mixed inflammation, and non-inflammatory patterns. IgE could induce senescence of smooth muscle cells in asthmatic model (Guo et al., 2019). TSLP could lead to bronchial epithelial cell senescence (Wu et al., 2013a). Neutrophil and Th17 inflammation is more likely correlated to steroid-resistant asthma (Israel and Reddel, 2017). IL-17 could enhance the secretion of SASP cytokines in bronchial fibroblasts, such as GM-CSF, TNF-α, IL-1β, and IL-6 (Molet et al., 2001). Vice versa, SASP associated factors (IL-1β, IL-6, IL-8, and GM-CSF) is closely associated with increase of both neutrophil and eosinophil inflammation (Busse et al., 2017). Third, although epithelial senescence-induced barrier dysfunction is also important in the pathogenesis of COPD and IPF, the mediator of epithelial cell senescence might be different in asthma. In IPF, we could find out a lot of gene mutations correlating to the premature aging of epithelial cells (Richeldi et al., 2017), while those gene mutations haven’t been reported in asthma. Instead, according to current studies, ITGB4 might be a pivotal gene to link epithelial cell senescence and the development of asthma (Liu et al., 2010a, b; Yuan et al., 2019), while it has not been demonstrated in COPD and IPF.

## Conclusion and Future Perspectives

**FIGURE 1:**
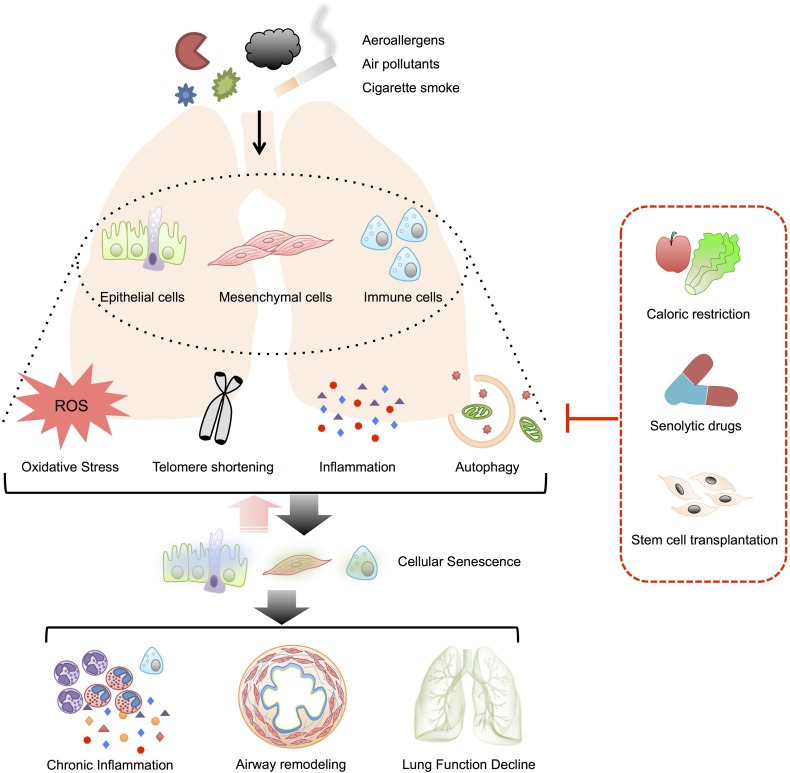
Possible role of cellular senescence in asthma. Telomere damage, oxidative stress, inflammation and insufficient/non-selective autophagy induced by various stimuli might mediate the senescence of airway epithelial cells, mesenchymal cells, and immune cells in asthmatics. The senescent cells could also augment surrounding cell senescence through SASP and form a vicious cycle. The senescence in these target cells would contribute to the pathobiology of asthma, including airway inflammation, airway remodeling as well as lung function decline. By the way of suspending the mediators of cellular senescence, anti-senescence strategies such as caloric restriction, senolytic drugs, and stem cells transplantation might serve as novel therapies for patients with asthma.

In this review, we summarized the current evidences illustrating the possible correlation of cellular senescence and the pathophysiology of asthma. We assume that asthma-related risk factors like invasive allergens, environmental pollutants or cigarette smoke could induce telomere shortening, oxidative stress, inflammation and insufficient/unselective autophagy, leading to the cellular senescence in epithelial cells, mesenchymal cells, and immune cells. Aging of these cells will then break the epithelial barrier, induce airway remodeling and sustain airway inflammation through SASP, which could augment cellular senescence in surrounding proliferating cells. Such a feedback loop promotes the pathogenesis of asthma. Thus, breaking this vicious cycle by anti-senescence strategies may help restrain the development of asthma (Figure 1).

Current understanding on the involvement of cellular senescence in asthma is hampered for several reasons. First, most of studies have neglected the detection of cellular senescence markers when investigating the role of telomere shortening, oxidative stress, inflammation and autophagy in the pathogenesis of asthma. Second, prospective data is limited so that it is difficult to figure out whether cellular senescence is a cause or a result of asthma. Third, few investigations have focused on the mechanisms of anti-senescence therapies for asthma. Thus, future studies need to put additional emphasis on ascertaining the place of cellular senescence in asthma by using and the mechanisms of anti-senescence therapies for patients with asthma. Sufficient methods for detecting premature senescence are necessary for future studies, including SA-β-Gal staining, proliferation assay, cell morphology, formation of senescence-associated heterochromatin foci, and secretion of SASP components.

## Author Contributions

Z-NW and R-NS contributed to the writing and revising of the manuscript. B-YY designed the figure and helped to draft the manuscript. K-XY and L-FY contributed to the searching of the related articles and reviews, and helped to draft the manuscript. YY provided knowledge on the molecular biology and critically revised the manuscript. Z-GC conceived the original idea and fixed the final outline. All authors read and approved the manuscript for publication.

## Conflict of Interest

The authors declare that the research was conducted in the absence of any commercial or financial relationships that could be construed as a potential conflict of interest. The handling Editor declared a past collaboration with one of the authors YY.
